# Combined systemic inflammation score (SIS) correlates with prognosis in patients with advanced pancreatic cancer receiving palliative chemotherapy

**DOI:** 10.1007/s00432-020-03361-0

**Published:** 2020-08-25

**Authors:** M. Markus, A. Abendroth, R. Noureddine, A. Paul, S. Breitenbuecher, I. Virchow, K. W. Schmid, P. Markus, B. Schumacher, M. Wiesweg, J. Wendling, B. Mende, J. T. Siveke, M. Schuler, S. Kasper

**Affiliations:** 1 West German Cancer Center, Department of Medical Oncology, University Hospital Essen, Hufelandstr. 55, 45147 Essen, Germany; 2 Department of Anesthesiology and Operative Intensive Care Medicine (CCMCVK), Charité – University Hospital Berlin, Berlin, Germany; 3 Department of Hematology, Oncology, and Clinical Immunology, Heinrich Heine University Düsseldorf, Düsseldorf, Germany; 4 West German Cancer Center, Department of General, Visceral and Transplant Surgery, University Hospital Essen, Essen, Germany; 5 Institute for Quality Assurance, University Hospital Essen, Essen, Germany; 6 West German Cancer Center, Institute of Pathology Essen, University Hospital Essen, Essen, Germany; 7 Department of General Surgery and Traumatology, Elisabeth Hospital Essen, Essen, Germany; 8 Department of Gastroenterology, Elisabeth Hospital Essen, Essen, Germany; 9 Central Pharmacy, University Hospital Essen, Essen, Germany; 10 German Cancer Consortium (DKTK), Partner Site University Hospital Essen, Essen, Germany; 11 West German Cancer Center, Institute for Developmental Cancer Therapeutics, University Hospital Essen, Essen, Germany; 12 Medical Faculty, University Duisburg-Essen, Essen, Germany

**Keywords:** Pancreatic cancer, Prognosis, Inflammation

## Abstract

**Purpose:**

The prognosis of patients with advanced pancreatic ductal adenocarcinoma (PDAC) remains dismal. New cytotoxic agents such as nab-paclitaxel and liposomal irinotecan (nal-Iri) have extended the armamentarium of therapeutic options in the last years. Nowadays, sequential therapeutic strategies with moderately toxic chemotherapeutic protocols can be administered to the patients. However, prognostic and predictive biomarkers are still missing to identify those patients, which profit most from a “continuum of care” concept rather than receiving intensive first-line protocols such as FOLFIRINOX. To this end, we retrospectively evaluated the impact of the systemic inflammation as one essential hallmark of cancer in patients with advanced PDAC treated with sequential systemic.

**Methods:**

A cohort of 193 PDAC patients treated at our center from January 2005 to August 2011 were retrospectively evaluated for the following systemic inflammatory response (SIR) markers: neutrophil–lymphocyte ratio (NLR), lymphocyte–monocyte ratio (LMR) C-reactive protein (CRP), and the modified Glasgow Prognostic Score (mGPS). SIR markers were correlated with clinico-pathological findings, response to chemotherapy and overall survival (OS) using Kaplan–Meier curves and Cox proportional models.

**Results:**

All evaluated SIR markers were significantly associated with OS in patients with metastatic disease but not in patients with locally advanced PDAC. Interestingly, all SIR markers were only prognostic in patients not receiving antibiotics as surrogate marker for systemic bacterial infections. Based on the evaluated SIR markers, we propose a new Systemic Inflammation Score (SIS), which significantly correlated with reduced OS (HR: 3.418 (1.802–6.488, *p* < 0.001)) and the likelihood of receiving further-line systemic therapies (*p* = 0.028).

**Conclusion:**

Routinely assessed SIR biomarkers have potential to support therapeutic decision making in patients with metastatic PDAC.

**Electronic supplementary material:**

The online version of this article (10.1007/s00432-020-03361-0) contains supplementary material, which is available to authorized users.

## Introduction

Pancreatic ductal adenocarcinoma (PDAC) has a rising incidence over the last decades and is the fourth leading cause of cancer-related death in Western countries (Saif 2013). Despite the implementation of new chemotherapeutic protocols like the combination of oxaliplatin, 5-fluoruracil, leucovorin and irinotecan (FOLFIRINOX) and the combination of gemcitabine and albumin-bound paclitaxel (nab-paclitaxel) in the palliative and adjuvant setting, the prognosis is still very poor with 5-year survival rates of 5–20% in patients with resectable tumors and < 5% in patients with locally advanced (LAPC) or metastatic disease (mPDAC) (Conroy et al. 2011, 2018; Von Hoff et al. 2013). Due to the aggressive tumor biology with intensive tumor stroma interaction, early metastasis and primary resistance to multiple cytotoxic drugs the treatment of patients with LAPC and mPDAC is very challenging. In addition, only a subgroup of patients with a good performance score, absent comorbidities and bilirubin levels within normal range will be eligible for the new cytotoxic combinations. Thus, a substantial number of patients will still receive gemcitabine monotherapy or only best supportive care. So far, no valid predictive or prognostic biomarkers have been established to better guide the systemic treatment of patients with advanced PDAC. Intra-tumoral inflammation is one essential hallmark of cancer initiation and progression through DNA damage and activation of intracellular signaling pathways (Hanahan and Weinberg 2011). This is supported by the fact that chronic pancreatitis is one of the major risk factors for the development of PDAC. The activation of the systemic immune system by the local intra-tumoral inflammation can be easily measured by blood-based parameters. These systemic inflammatory response (SIR) parameters such as the C-reactive protein (CRP) or different levels of white blood cells and their respective ratios such as the neutrophil–lymphocyte (NLR) ratio and others has been previously correlated with the risk of recurrence and overall survival in curatively or palliatively treated malignancies including PDAC (Hang et al. 2017; Martin et al. 2014; Proctor et al. 2011; Schlick et al. 2019; Stotz et al. 2013, 2015). However, data in PDAC are controversial, most investigators did not stratify the patients based on the extent of disease and used different clinical endpoints and cut-offs for the investigated SIR markers (Jamieson et al. 2011; Singh et al. 2017; Wang et al. 2012). In addition, most studies focused only on one SIR marker and did not use a combination of different markers for a better dissection of patients’ prognosis.

Against this background, the aim of the study was the investigation of already published prognostic SIR markers in patients with LAPC and mPDAC with mature follow-up, which received palliative chemotherapy in the era before the broad clinical implementation of nab-paclitaxel and FOLFIRINOX. Here, we investigated the prognostic value of the lymphocyte-monocyte ratio (LMR), the neutrophil–lymphocyte ratio (NLR), the modified Glasgow Prognostic Score (mGPS) and the CRP in a cohort of 193 patients with PDAC treated at the one single comprehensive cancer center. Based on our findings, we proposed a new combination Systemic Inflammation Score (SIS), which could help in clinical decision making. In addition, this SIS could serve as stratification factor for future randomized clinical trials to ensure allocation of patients with good or poor prognosis to each experimental treatment arm. Furthermore, it should be investigated in a prospective clinical trial, if poor prognosis patients based on the proposed SIS will benefit from a more intensive first-line chemotherapy.

## Methods

### Study design

Patients with histological confirmed LAPC or mPDAC diagnosed between January 2005 and August 2011 were retrospectively enrolled into this biomarker analyses. Resected patients with relapse or metachronous metastases were included at the time point of first palliative chemotherapy. Chemotherapy regime was selected by the treating oncologist based on the performance status, comorbidities and patient wish. Follow-up were routinely assessed and documented in the electronic health record (EHR). Clinical parameters, applied chemotherapy protocols including efficacy data were also retrieved from the EHR. Laboratory values including absolute neutrophil count, absolute lymphocyte count, absolute monocyte count, C-reactive protein (CR), albumin, direct bilirubin and carboanhydrate antigen 19-9 (CA19-9) were assessed before the start of palliative chemotherapy. The use of antibiotics was also retrieved from the EHR. Personal patient data were anonymized in the data base and the data were analyzed by a blinded researcher. The study was approved by the Ethics Committee of the Medical Faculty of the University Duisburg-Essen (Project No. 15-6497).

### Assessments

Tumor staging was performed according to the American Joint Committee on Cancer (AJCC)/International Union against Cancer (UICC) TNM classification (7th Edition). Clinical staging was routinely based on computed tomography (CT) or magnetic resonance imaging (MRI) before start of palliative chemotherapy and subsequently every 8–12 weeks. Overall response rate (ORR) was evaluated according to the Response Evaluation Criteria in Solid Tumors 1.1 (RECIST 1.1). Patients were eligible for ORR assessment if they had a baseline radiological examination and at least one examination during the palliative chemotherapy. ORR was defined as the proportion of patients with complete or partial remission, the disease control rate (DCR) was defined as the proportion of patients with complete or partial remission or sustained disease stabilization). Progression-free survival (PFS) was defined as time from start of chemotherapy to date of radiological or clinical progression or death. Overall survival was defined as time from start of palliative therapy to death. Patients were censored at the time of last visit at our center, if time of death was not evaluable.

### Statistical analysis

All analyses were conducted using SPSS Statistics (V19, IBM, Armonk, NY, USA). Correlation analyses were performed using Spearman-Rho- or Pearson’s Chi-square test. Kaplan–Meier calculations with the log rank test were used for analysis of OS and PFS. Univariate analyses were performed by a Cox proportional-hazard model. Hazard ratio (HR) and 95% confidence intervals (CI) were indicated. Overall, *p* values ≤ 0.05 were regarded statistically significant.

## Results

### Patients’ characteristics

In total, 859 patients with PDAC diagnosed between January 2005 and August 2011 were identified in the database of our hospital. A substantial number of patients did not have their primary diagnosis at our center, had no advanced disease or the follow-up was missing. Thus, from this cohort, a total of 193 patients (53.4% male) with LAPC or mPDAC with a sufficient follow-up were enrolled into this retrospective analysis (suppl. Fig. 1).

The median follow-up time was 0.135.0 months (range 98.9–232.2 months). Patients’ characteristics are summarized in Table 1. Median age was 69 years (range 31–89 years); 40.9% of patients were older than 65 years and 18.1% of patients were older than 70 years. Most patients had metastatic disease (79.8%, mPDAC), 20.2% of patients had locally or locally advanced pancreatic cancer (LAPC). The most prevalent metastatic sites were liver (56.5%), lymph nodes (23.8%), lung (10.6%) and the peritoneum (22.3%). In total, 24 patients (12.4%) underwent surgery in curative intent and 32 patients (16.6%) received a palliative surgery. Placement of a stent for management of biliary obstruction was required in 10.9% of patients. Adjuvant/Additive chemotherapy was administered in 79.2% of curatively resected patients. The median disease free survival of curatively resected patients was 13 months (95% CI 10.1–15.9).

**Table 1:** Baseline clinical characteristics (*N* = 193)

Gender	Female	46.6%
Median age	63 years (range 31–89)	
Age > 65 years	40.9%	
Age > 70 years	18.1%	
Stage at diagnosis	Locally/locally advanced Metastatic	20.2% 79.8%
Primary location	Head	58.0%
	Body	20.2%
	Tail	16.1%
	Multiple sides	3.6%
	Unknown	2.1%
Histology	Adenocarcinoma	97.9%
	Other (*N* = 4)	2.1%
Grading	G1	3.1%
	G2	45.6%
	G3	18.7%
	Unknown*	32.6%
Bilirubin > 1.5ULN	16.1%	
Initial CA 19–9 (median)	565 U/ml (range 1–463, 600U/ml)	
Primary resection in curative intent	*N* = 24	12.4%
	R0	23.2%
	R1	17.9%
	R2	1.8%
	Rx	57.2%
Adjuvant/additive chemotherapy	19	79.2% of curative resected
Palliative surgery	*N* = 32	16.6%
Primary stenting	*N* = 21	10.9%
Median disease free survival after resection (95% CI)		13 months (10.1–15.9)
Sites of metastasis	Liver	56.5%
	Lymph nodes	23.8%
	Lung	10.9%
	Peritoneum	22.3%
	Other	14.5%
Follow-up time (range)	135.0 months (98.9–232.2)	
Number of cases lost to follow-up	*N* = 21 (10.9%)	
Number of death	N = 171 (88.6%)	

*ULN* upper limit of normal, *CA 19–9* carbohydrate antigen 19–9, *CI* confidence interval

*Diagnosis by cytology

At time point of palliative chemotherapy, baseline serum bilirubin was elevated (> 1.5 ULN) in 16.1% of patients; median carboanhydrate antigen 19–9 (CA 19–9) level was 565 U/ml, range 1–463,600 U/ml). At the time point of data cut-off (05.11.2019), 171 patients (88.6%) were dead, 21 patients were lost to follow-up and 1 patient was still alive.

### Efficacy of palliative chemotherapy

**Fig. 1:**
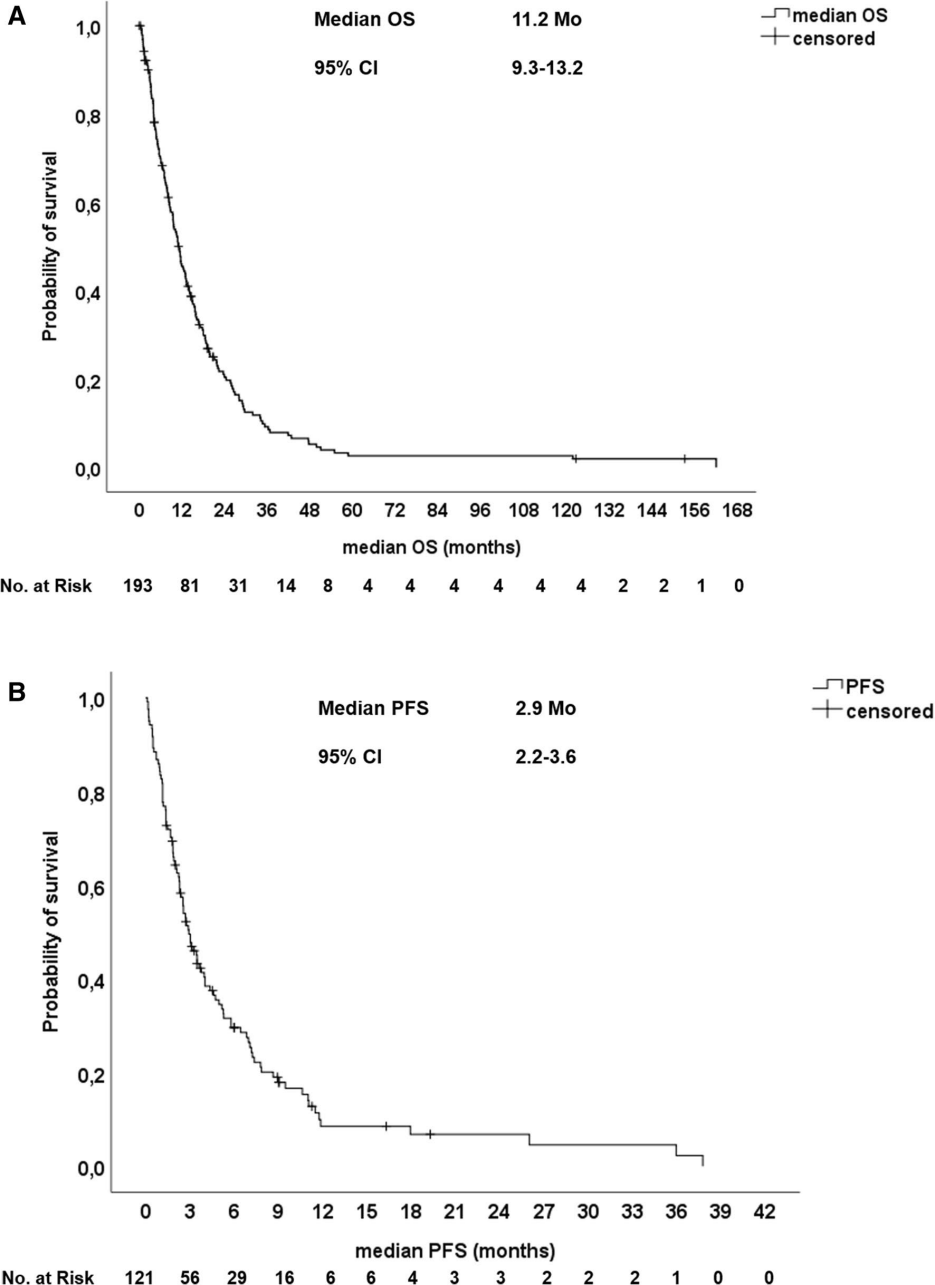
Kaplan-Meier-plot for (**a**) Overall survival (OS) and (**b**) Progression-free survival (PFS) of the entire patient population upon first-line chemotherapy

The majority of patients (54.4%) received a first-line systemic palliative monotherapy with gemcitabine, 26.4% of patients were treated with a doublet chemotherapy of gemcitabine in combination with oxaliplatin or cisplatin and 19.2% of patients received combination therapies with fluoropyrimidines or gemcitabine in combination with erlotinib (Table 2). In total, 157 patients (81.3%) were evaluable for response assessment according to RECIST 1.1. The ORR of first-line therapy was 10.9%, the DCR was 31.1% and 50.3% of patients had progressive disease upon first-line palliative chemotherapy. The median PFS upon first-line therapy was 2.9 months. After failure of first-line therapy, 47.2% of patients received a second-line therapy and 25.9% received three or more lines of therapy. The median PFS upon 2nd and 3rd line therapy were 3.2 and 2.1 months, respectively. The median OS from start of palliative treatment was 11.2 months for the entire population (Fig. 1a, b). Patients with locally/locally advanced disease (LAPC) had a significant longer median OS of 26.4 months compared to patients with metastatic disease (mPDAC) with a median OS of only 9.4 months (Fig. 3a).

**Table 2:** Outcome upon palliative chemotherapy

Systemic therapy		
Monotherapy	*N* = 105	54.4%
Platinum doublet	*N* = 51	26.4%
Other combination	*N* = 37	19.2%
Number therapy lines (median)	2 range (1–4)	
2nd line received	91	47.2%
3rd line received	50	25.9%
4th line received	20	10.4%
CR	*N* = 2	1.0%
PR	*N* = 19	9.8%
SD	*N* = 39	20.2%
PD	*N* = 97	50.3%
n.e	*N* = 36	18.7%
Overall response rate (ORR)	*N* = 21	10.9%
Disease control rate (DCR)	*N* = 60	31.1%
Median PFS of 1st line CTX	2.9 months	(95% CI 2.2–3.6 months)
Median PFS of 2nd line CTX	3.2 months	(95% CI 2.0–4.3 months)
Median PFS of 3rd line CTX	2.1 months	(95% CI 1.7–2.4 months)
Median PFS of 4th line CTX	1.8 months	(95% CI 1.6–2.0 months)
Median OS entire population	11.2 months	(95% CI 9.3–13.2 months)
Median OS locally/locally advanced	26.4 months	(95% CI 20.3–30.6 months)
Median OS metastatic disease	9.4 months	(95% CI 7.5–11.2 months)

*PFS* progression-free survival, *OS* overall survival

### Systemic inflammatory response (SIR) parameter as explorative prognostic markers

**Fig. 2:**
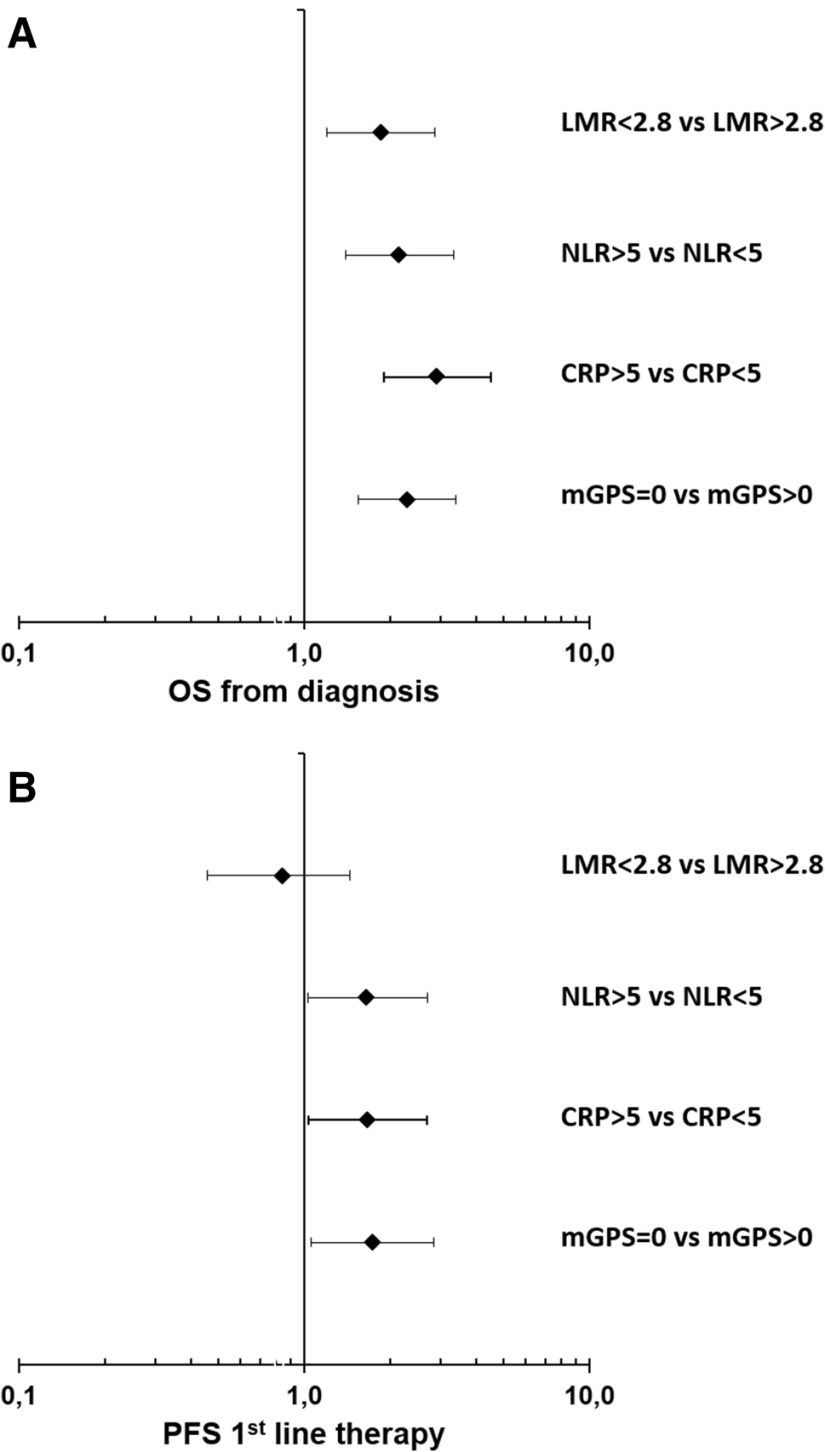
**a, b** Forest plot of hazard rations (HR) for **a** Overall survival (OS) and **b** Progression-free survival (PFS) including lymphocyte–monocyte ratio (LMR), neutrophil–lymphocyte ratio (NLR), C-reactive protein (CRP) and modified Glasgow Prognostic Score (mGPS)

**Fig. 3:**
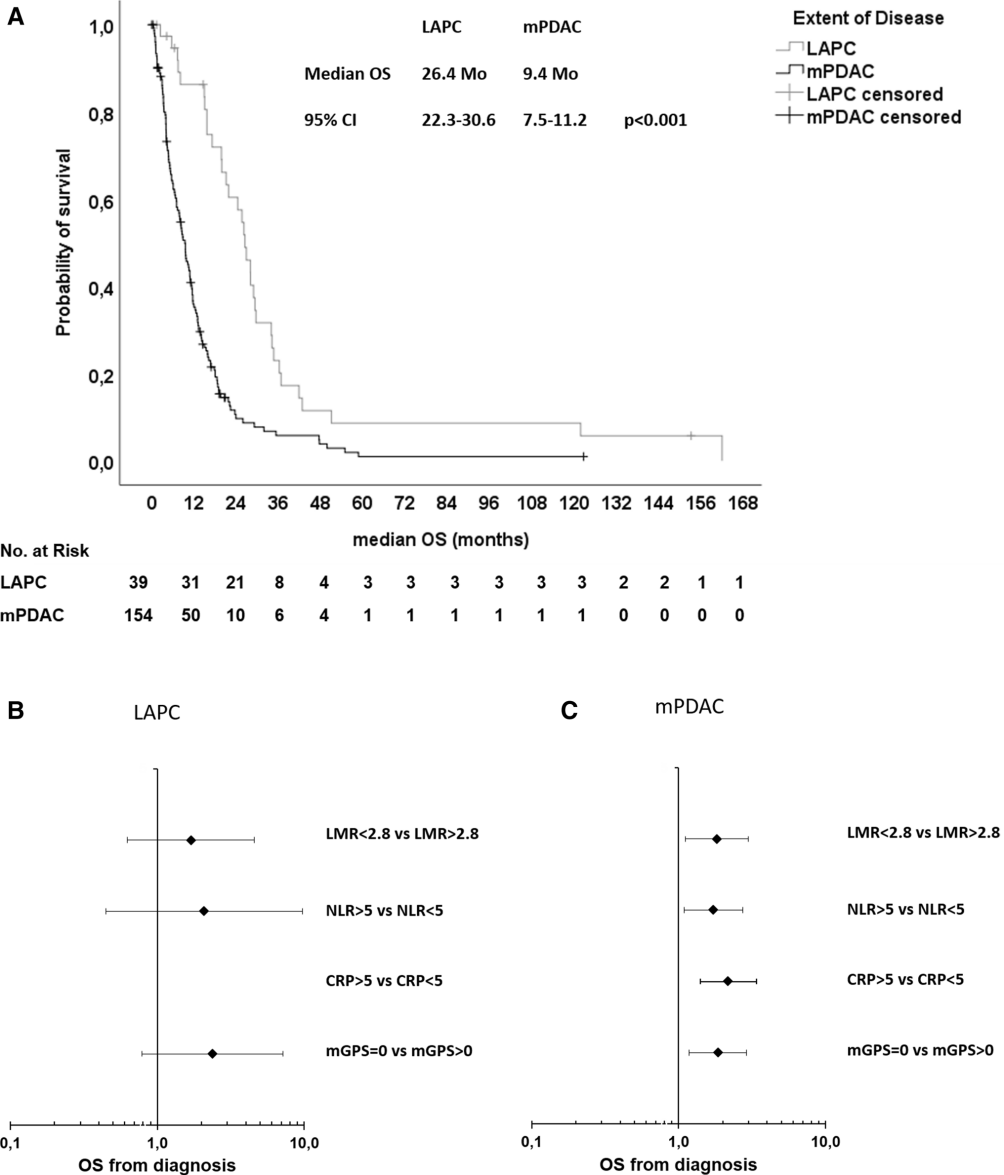
**a** Kaplan–Meier plot for (**a**) overall survival (OS) upon first-line palliative chemotherapy in relation to the extent of disease. **a** Patients with locally advanced pancreatic cancer (LAPC) had a median OS of 26.4 months and patients with metastatic pancreatic ductal adenocarcinoma (mPDAC) a median OS of 9.4 months (p < 0.001, log rank). **b** Forest plot of hazard rations (HR) for Overall survival (OS) for patients with locally advanced pancreatic cancer (LAPC) including lymphocyte–monocyte ratio (LMR), neutrophil–lymphocyte ratio (NLR), C-reactive protein (CRP) and modified Glasgow Prognostic Score (mGPS). **c** Forest plot of hazard rations (HR) for Overall survival (OS) for patients with metastatic pancreatic ductal adenocarcinoma (mPDAC) including lymphocyte-monocyte ratio (LMR), neutrophil–lymphocyte ratio (NLR), C-reactive protein (CRP) and modified Glasgow Prognostic Score (mGPS)

We assessed the following markers of systemic inflammatory response (SIR): lymphocyte-monocyte ratio (LMR), neutrophil–lymphocyte ratio (NLR), c-reactive protein (CRP), and the modified Glasgow prognostic score (mGPS)—a composite marker of high CRP-levels (> 1 mg/dl) and low albumin-levels (< 3.5 g/dl) (Proctor et al. 2011). All parameters were assessed before the administration of the first palliative treatment. For the LMR and NLR, data from 108 patients were available, for CRP and the mGPS, data from 120 patients were available (Table 3, suppl. Fig. 1). For the explorative analyses, we chose cut-off values listed in Table 3. These cut-off values were previously published as prognostic markers in different malignancies including pancreatic cancer (Hang et al. 2017; Martin et al. 2014; Stotz et al. 2013, 2015). In our patient cohort, 69.4% had a LMR < 2.8, 31.5% had a NLR > 5, 26.7% had a CRP > 5 mg/dl and 62.5% had an mGPS > 1. First, we correlated the SIR parameters with established negative prognostic markers: grading, metastatic disease (M1) and elevated CA19-9 levels (suppl. Table 1). We found that all SIR markers correlated with at least two known negative prognostic markers. Next, we studied the impact of the SIR markers on treatment outcomes. All evaluated markers significantly correlated with the median OS upon first-line palliative chemotherapy (Table 4; Fig. 2a; suppl. Figs. 2 A, 3 A, 4 A, 5 A). Patients with a LMR > 2.8 had a median OS of 12.8 months compared to patients with a LMR < 2.8 of only 7.7 months, patients with a NLR < 5 had a median OS of 9.8 months compared to patients with a NLR > 5 of only 4.8 months, patients with a CRP < 5 mg/dl had a median OS of 10.8 months compared to patients with a CRP > 5 mg/dl of only 3.9 months and patients with a mGPS of 0 had a median OS of 13.3 months compared to patients with a mGPS > 0 of only 5.2 months. In line, all SIR markers except the LMR correlated with the median PFS of first-line systemic therapy (Table 4; Fig. 2b; suppl. Figs. 2 B, 3 B, 4 B, 5 B). Patients with LAPC had a significantly longer median OS than patients with mPDAC (Fig. 3a). To test if the SIR markers were prognostic in both subgroups, we next stratified the patients by extent of disease and correlated the SIR markers with the OS (Table 5). Interestingly, all SIR markers only correlated with the median OS in patients with metastatic disease and not in patients with LAPC (Fig. 3b, c).

**Table 3:** SIR marker

Marker	Patients with data	Cut-off	Patients < cut-off (%)	Patients > cut-off (%)
LMR	108	2.8	75 (69.4%)	33 (30.6%)
NLR	108	5	74 (68.5%)	34 (31.5%)
CRP	120	5 mg/dl	88 (73.3%)	32 (26.7%)
mGPS	120	1	45 (37.5%)	75 (62.5%)

*LMR* lymphocyte–monocyte ratio, *NLR* neutrophil–lymphocyte ratio, *CRP* C-reactive protein, *mGPS* modified Glasgow Prognostic Score

**Table 4:** Outcome in SIR subgroups

	Median OS				Median PFS 1st line			
	Months (95% CI)	*p*	HR (95% CI)	*p*	Months (95% CI)	*p*	HR (95% CI)	*p*
LMR > 2.8 < 2.8	12.8 (9.0–16.7) 7.7 (5.0–10.4)	0.005	1.860 (1.201–2.880)	0.005	2.5 (1.9–3.1) 2.8 (1.6–3.9)	0.521	0.838 (0.458–1.445)	0.524
NLR < 5 > 5	9.8 (7.8–11.7) 4.8 (3.4–6.2)	< 0.001	2.164 (1.397–3.351)	0.001	2.9 (1.5–4.3) 2.6 (0.6–4.5)	0.041	1.653 (1.013–2.697)	0.044
CRP < 5 mg/dl > 5 mg/dl	10.8 (9.1–12.4) 3.9 (2.8–5.0)	< 0.001	2.935 (1.904–4.526)	< 0.001	3.5 (1.3–5.0) 1.2 (0.0–2.3)	0.034	1.671 (1.033–2.704)	0.036
mGPS 0 > 0	13.3 (8.2–18.4) 5.2 (3.5–7.0)	< 0.001	2.297 (1.545–3.414)	< 0.001	5.8 (4.5–7.1) 2.3 (1.1–3.5)	0.027	1.738 (1.056–2.861)	0.030

*LMR* lymphocyte-monocyte ratio, *NLR* neutrophil–lymphocyte ratio, *CRP* C-reactive protein, *mGPS* modified Glasgow Prognostic Score, *M1* metastatic disease

**Table 5:** Impact of SIR markers in patients with LAPC and mPDAC

	LAPC				mPDAC			
	Months (95% CI)	*p*	HR (95% CI)	*p*	Months (95% CI)	*p*	HR (95% CI)	*p*
All	26.4 (22.3–30.6)	< 0.001	2.693 (1.816–3.992)	< 0.001	9.4 (7.5–11.2)			
LMR > 2.8 < 2.8	19.7 (4.8–34.7) 21.1 (11.9–30.3)	0.291	1.695 (0.626–4.589)	0.299	9.7 (8.3–11.3) 5.4 (2.9–7.8)	0.016	1.816 (1.111–2.970)	0.017
NLR < 5 > 5	21.1 (18.5–23.7) 7.3 (n.a.)	0.333	2.093 (0.449–9.746)	0.347	8.7 (7.1–10.3) 4.7 (3.7–5.8)	0.018	1.724 (1.090–2.725)	0.020
CRP < 5 mg/dl > 5 mg/dl	21.1 (18.2–23.9) n.a	n.a	n.a	n.a	9.5 (8.2–10.9) 3.9 (2.8–5.0)	< 0.001	2.174 (1.401–3.376)	0.001
mGPS 0 > 0	21.1 (12.8–29.4) 15.0 (0.0–33.3)	0.112	2.376 (0.788–7.167)	0.124	10.8 (8.8–12.8) 5.1 (3.5–6.7)	0.007	1.849 (1.178–2.901)	0.008

*LMR* lymphocyte-monocyte ratio, *NLR* neutrophil–lymphocyte ratio, *CRP* C-reactive protein, *mGPS* modified Glasgow Prognostic Score, *LAPC* locally advanced pancreatic cancer, *mPDAC* metastatic pancreatic ductal adenocarcinoma

### Impact of infections on SIR markers and prognosis

**Fig. 4:**
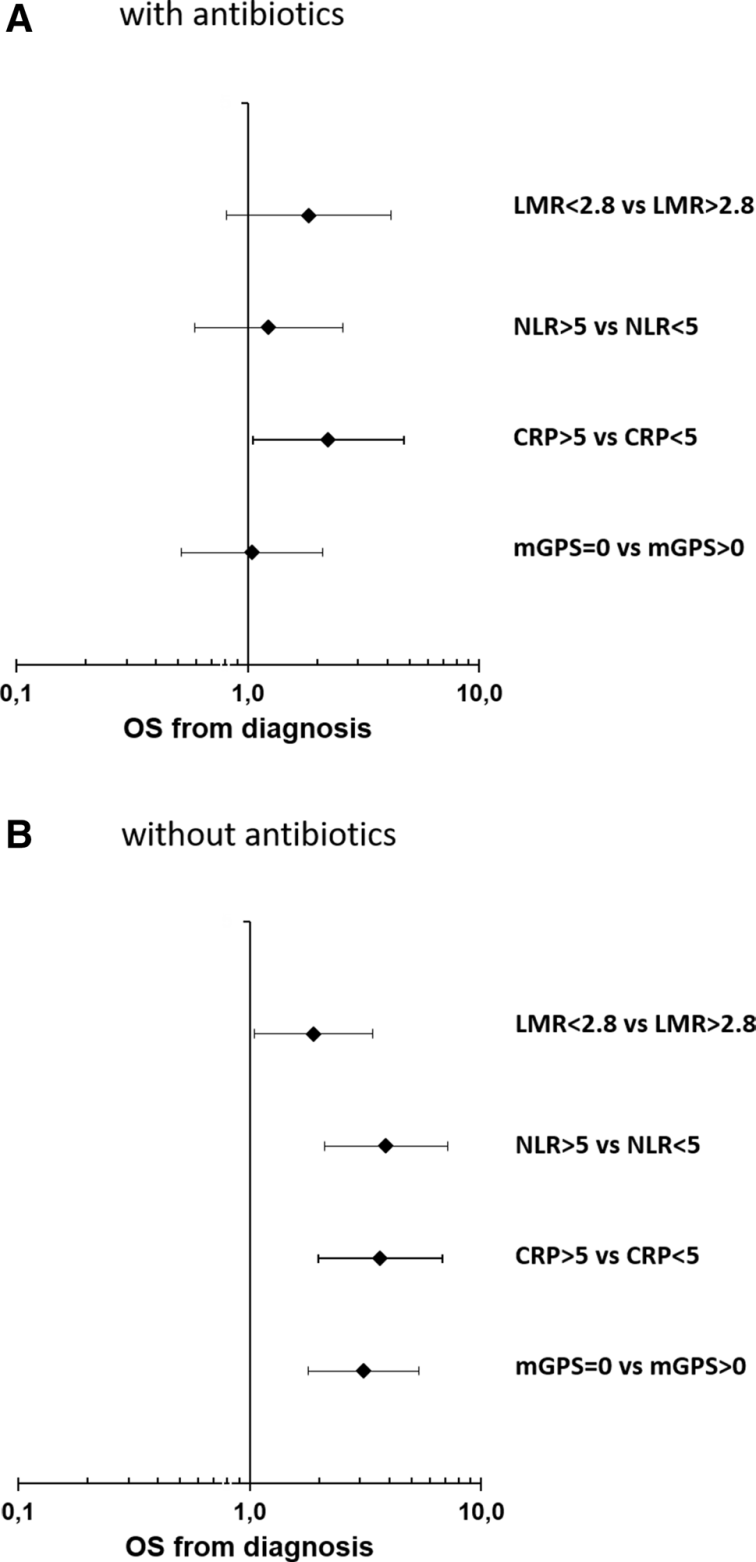
Forest plot of hazard rations (HR) for Overall survival (OS) for patients which received antibiotics (**a**) and patients without antibiotics (**b**) including lymphocyte–monocyte ratio (LMR), neutrophil–lymphocyte ratio (NLR), C-reactive protein (CRP) and modified Glasgow Prognostic Score (mGPS)

Systemic inflammatory response could not only be associated with an aggressive tumor behavior but could also be a sign of systemic infections. In particular, patients with pancreatic cancer often suffer from systemic and biliary tract infection due to cancer-induced immunosuppression and malignant biliary obstruction (Plate and Harris 2000). To exclude confounding of our observation by the presence of active bacterial infections, which may themselves associate with inferior outcome, we next stratified our cohort according to the presence or absence of antibiotic therapy during chemotherapy for PDAC. Information on the use of antibiotics could be collected from the EHR in 148 patients, of which 53 patients (35.8%) received antibiotics. We did not observe a significant difference in median OS between patients with or without antibiotic therapy (10.8 months vs. 11.3 months) (Suppl. Fig. 6). Interestingly, all analyzed SIR markers correlated with a poor prognosis only in patients without antibiotic therapy (Fig. 4b). In patients receiving antibiotics, only elevated CRP > 5 mg/dl correlated with inferior OS, whereas LMR, NLR and the mGPS had no impact on the prognosis (Fig. 4a). In conclusion, SIR markers strongly associate with prognosis in patients with metastatic PDAC without clinical signs of bacterial infection. In patients receiving antibiotics, their prognostic impact is confounded by infection-related modulation of SIR markers.

### Systemic inflammation score (SIS)

**Fig. 5:**
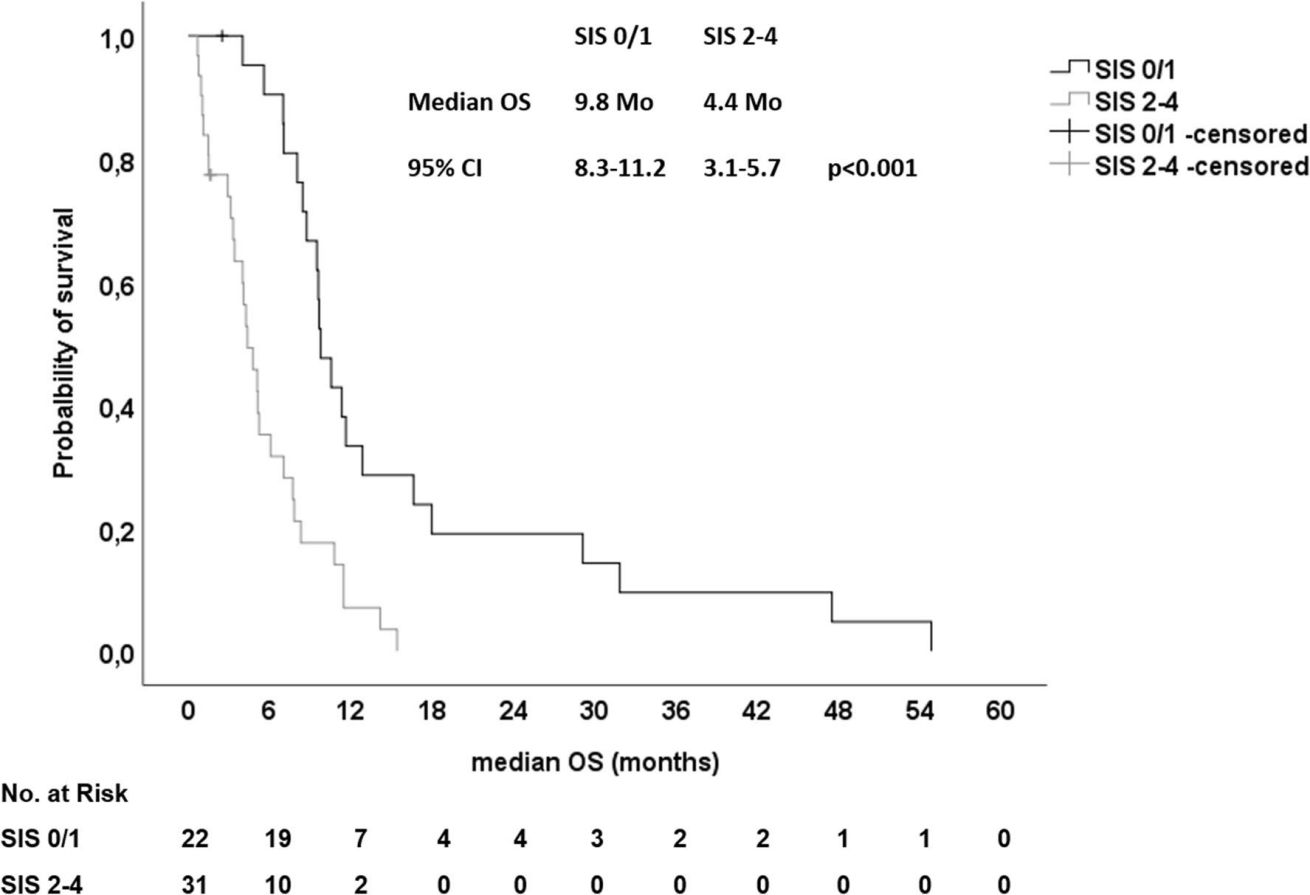
Kaplan–Meier plot for overall survival (OS) in patients with metastatic disease, which were not treated with antibiotics upon first-line palliative chemotherapy stratified for the Systemic Inflammation Score (SIS). Patients with a SIS of 0/1 had a median OS of 9.8 months and patients with a SIS of 2–4 had a median OS of only 4.4 months (*p* < 0.001, log rank)

For further assignment patients with pancreatic cancer to different prognostic risk groups, we proposed a new Systemic Inflammation Score (SIS) based on the four identified prognostic SIR markers. For each positive SIR marker, one point was allocated (LMR < 2.8, NLR > 5, CRP > 5 mg/dl and mGPC > 0). We excluded patients with locally advanced disease and patients with antibiotic therapy for this analysis. First, we assigned our patients into four groups (0, 1, 2, 3 and 4 points) and correlated the different groups with the median OS (Table 6). Patients with a SIS of 0 and 1 had a relative favorable prognosis with a median OS of 10.5 and 9.6 months, respectively. In contrast, patients with a SIS of 2, 3 and 4 had a significantly shorter median OS of 5.2, 4.8 and 1.5 months, respectively (*p* < 0.001) (Suppl. Fig. 7). Hence, we grouped our patients into a group with favorable prognosis (SIS 0 and 1) and into a group of patients with a poor prognosis (SIS > 1). Patients with a SIS of 0 and 1 had a median OS of 9.8 months and patients with a SIS > 1 had a significantly shorter median OS of only 4.4 months (*p* < 0.001) (Table 6 and Fig. 5). In addition in the entire cohort, 59.1% of patients with a SIS of 0 and 1 received second- and further-line therapies, whereas only 40.9% of patients with a SIS > 1 received an additional therapy line after failure of first-line therapy. Thus, patients with a high SIS had a significantly lower chance to receive more than one line of systemic palliative chemotherapy (*p* = 0.028; Pearson’s Chi-square). In conclusion, we propose a novel SIS, which could easily assign patients with PDAC treated with systemic chemotherapy into prognostic risk groups and could serve as stratification factor for future randomized clinical trials. In addition, it should be investigated if patients with a high SIS will benefit from a more intensive first-line chemotherapy rather than sequential therapy strategies.

**Table 6:** median OS in patients based on Systemic Inflammation Score (SIS) (N = 53)

	*N*	%	Months (95% CI)	*p*	HR (95% CI)	*p*
SIS						
0	10	18.9	10.5 (8.2–12.9)	n.a	1	n.a
1	12	22.6	9.6 (9.3–9.8)	0.176	1.148 (0.466–2.829)	0.764
2	11	20.8	5.2 (3.4–7.1)	< 0.001	1.805 (1.088–2.994)	0.022
3	13	24.5	4.8 (1.6–8.0)	0.003	1.414 (1.011–1.978)	0.043
4	7	13.2	1.5 (0.3–2.7)	< 0.001	4.429 (0.609–32.236)	0.142
Combination						
0 + 1	22	41.5	9.8 (8.3–11.2)	< 0.001	3.419 (1.802–6.488)	< 0.001
2 − 4	31	58.5	4.4 (3.1–5.7)			

*OS* Overall Survival, *SIS* Systemic Inflammation Score, *n.a.* not applicable

## Discussion

The systemic palliative treatment of patients with PDAC remains challenging. Despite some progress in the last years with the implementation of new cytotoxic combinations therapies, the prognosis is still very limited. However, first-line cytotoxic combinations are not appropriate for patients with poor ECOG performance status, multiple comorbidities or elevated bilirubin levels. For these patients, gemcitabine monotherapy is still recommended. After failure of gemcitabine based first-line therapy, effective second- and further-line therapies have been established in large clinical trials. The combination of oxaliplatin and 5-FU (OFF) or nanoliposonal-irinotecan and 5-FU (NAPOLI) showed clinical effectivity and could prolong OS in this setting (Oettle et al. 2014; Wang-Gillam et al. 2016). Thus, nowadays, the paradigm of the systemic palliative treatment in PDAC has changed into a “continuum of care” concept with the possibility to sequentially administer cytotoxic therapies. Previously, we could demonstrate that the administration of sequential therapies with less toxic chemotherapeutic protocols could be an alternative to an intensive first-line treatment with comparable long-term OS in patients with advanced PDAC (Abendroth et al. 2019). However, predictive and prognostic biomarkers are still missing and are urgently needed to identify patients with the need of an intensive first-line chemotherapy or which could be treated with alternative, less toxic therapeutic strategies.

Here, we addressed the prognostic value of systemic inflammation and in particular on the easily measurable, blood-based SIR markers LMR, NLR, CRP and the mGPS in patients with advanced PDAC treated with systemic palliative chemotherapy. We correlated the clinical outcome of 193 patients with LAPC or mPDAC irrespective of age, co-morbidites or performance status treated with systemic palliative chemotherapy before the approval and routine implementation of modern chemotherapy protocols such as nab-paclitaxel or FOLFIRINOX. The majority of our patients received first-line monotherapy with gemcitabine, combination therapies of gemcitabine with platinum agents (cisplatin or oxaliplatin) or fluoropyrimidin-based combinations with irinotecan or oxaliplatin. The ORR of first-line therapy was 10.9%, which was comparable with the results published in the pivotal Burris trial with gemcitabine monotherapy or the gemcitabine control arms in the Prodige4/ACCORD11 and MPACT studies (Burris et al. 1997; Conroy et al. 2011; Von Hoff et al. 2013). Despite the moderate ORR and the PFS of only 2.9 months in our cohort, the median OS from start of palliative treatment was unexpectedly high with 11.2 months for the entire population and 9.4 months for patients with metastatic disease. Of note, nearly half of our patients received second- and further-line treatment, which could explain the unexpectedly favorable median OS. In line, patients, who received more than one line of therapy had a significant longer median OS of 15.0 months (95% CI 12.0–17.9) compared to only 7.0 months (95% CI 4.8–9.3) for patients treated with only one line of therapy (*p* = 0.004, log rank) (*data not shown*). To identify those patients, who profit most from a “continuum of care” with the sequential application of different, effective and less toxic chemotherapeutic protocols, we focused on easily, in the routine diagnostic assessable blood-based biomarkers of systemic inflammation. We chose cut-off values for all SIR markers which were previously published in different malignancies including pancreatic cancer (Hang et al. 2017; Martin et al. 2014; Stotz et al. 2013, 2015). However, we did not only focus on one or two markers as most studies before, but chose a comprehensive set of four SIR markers to better assign our patients into different risk groups. All our analyzed biomarkers of systemic inflammation (LMR, NLR, CRP and mGPS) correlated with a poor prognosis in our entire cohort. However, the negative impact of these SIR markers was restricted to patients with metastatic disease rather than to patients with LAPC. This could explain the controversial results with some of our used SIR markers in previous studies, which included mainly LAPC and not PDAC (Jamieson et al. 2011; Smith et al. 2009; Wang et al. 2012). In a large meta-analysis conducted by Yang and colleagues, the NLR significantly correlated with the prevalence of distant metastases in advanced PDAC (Yang et al. 2015). In line, the secretion of pro-inflammatory cytokines by tumor and immune cells promotes extravasation and metastasis of pancreatic carcinoma cells (Huang et al. 2017). Thus, systemic inflammation seems to have a major impact in patients with advanced metastatic stage rather than in patients with local or locally advanced disease.

To distinguish between tumor- or infection-mediated systemic inflammation response, we grouped our patients based on the use of antibiotics during the palliative chemotherapy. We found that the SIR markers were only prognostic in patients without antibiotics and without signs of systemic infection. To our knowledge, this is the first study, which analyzed the impact of antibiotic use in the interpretation of elevated SIR markers in PDAC or other malignancies. These results should be considered in future studies evaluating the impact of elevated SIR markers.

Finally, we proposed a new, easily applicable Systemic Inflammation Score (SIS) based on our four analyzed SIR markers to better assign patients with advanced PDAC to different prognostic risk groups. We could clearly identify a subgroup of patients with a SIS > 1 with dramatically reduced median OS. In addition, these patients were less likely to receive second- and further-line therapies (*p* = 0.028; Pearson`s Chi-square) and should be considered for intensive first-line therapeutic strategies rather than sequential therapies. This is in line with previous studies, which investigated the impact of NLR and CRP in patients with PDAC receiving palliative chemotherapy (Schlick et al. 2019).

We think the new proposed SIS could help to assign patients with advanced PDAC into different prognostic risk groups and could be a support for therapeutic decision. However, due to the limitation of our retrospective analyses, we suggest to validate our results prospectively in larger clinical trials.

## Electronic supplementary material

Below is the link to the electronic supplementary material.

Supplementary file1 (DOCX 12 kb)

Supplementary file2 (DOCX 14 kb)

Supplementary file3 (DOCX 14 kb)

Supplementary Figure 1 CONSORT Diagram. PDAC: pancreatic ductal adenocarcinoma; LAPC: locally advanced pancreatic cancer, mPDAC: metastatic pancreatic ductal adenocarcinoma; CRP: C-rective protein; mGPS: modified Glasgow Prognostic Score; NLR: neutrophil-lymphcyte ratio; LMR: lymphocyte-monocyte ratio (TIF 121 kb)

Supplementary Figure 2 A, B Kaplan-Meier-plot for (A) overall survival (OS) and (B) Progression-free Survival (PFS) upon first-line palliative chemotherapy in relation to the lymphocyte-monocyte ratio (LMR). A) Patients with a LMR <2.8 had a median OS of 7.7 months and patients with a LMR>2.8 had a median OS of 12.8 months (p=0.005, log rank). B) Patients with a LMR <2.8 had a median PFS of 2.8 months and patients with a LMR>2.8 had a median PFS of 2.5 months (p=0.524, log rank) (TIF 216 kb)

Supplementary file6 (TIF 214 kb)

Supplementary Figure 3 A, B Kaplan-Meier-plot for (A) overall survival (OS) and (B) Progression-free Survival (PFS) upon first-line palliative chemotherapy in relation to the neutrophil-lymphocyte ratio (NLR). A) Patients with a NLR>5 had a median OS of 4.8 months and patients with a NLR<5 had a median OS of 9.8 months (p<0.001, log rank). B) Patients with a NLR>5 had a median PFS of 2.6 months and patients with a NLR<5 had a median PFS of 2.9 months (p=0.044, log rank) (TIF 213 kb)

Supplementary file8 (TIF 206 kb)

Supplementary Figure 4 A, B Kaplan-Meier-plot for (A) overall survival (OS) and (B) Progression-free Survival (PFS) upon first-line palliative chemotherapy in relation to the C-reactive protein (CRP). A) Patients with a CRP>5 had a median OS of 3.9 months and patients with a CRP<5 had a median OS of 10.8 months (p<0.001, log rank). B) Patients with a CRP>5 had a median PFS of 1.2 months and patients with a CRP<5 had a median PFS of 3.2 months (p=0.036, log rank) (TIF 222 kb)

Supplementary file10 (TIF 215 kb)

Supplementary Figure 5 A, B Kaplan-Meier-plot for (A) overall survival (OS) and (B) Progression-free Survival (PFS) upon first-line palliative chemotherapy in relation to the modified Glasgow Prognostic Score (mGPS). A) Patients with a mGPS>0 had a median OS of 5.2 months and patients with a mGPS=0 had a median OS of 13.3 months (p<0.001, log rank). B) Patients with a mGPS>0 had a median PFS of 2.3 months and patients with a mGPS=0 had a median PFS of 5.8 months (p=0.030, log rank) (TIF 218 kb)

Supplementary file12 (TIF 212 kb)

Supplementary Figure 6 Kaplan-Meier-plot for overall survival (OS) upon first-line palliative chemotherapy in relation to the use of antibiotics. Patients, which received antibiotics had a median OS of 10.8 months and patients without antibiotics (w/o) had a median OS of 11.3 months (p=0.415, log rank) (TIF 224 kb)

Supplementary Figure 7 Kaplan-Meier-plot for overall survival (OS) in patients with metastatic disease, which were not treated with antibiotics upon first-line palliative chemotherapy stratified for the Systemic Inflammation Score (SIS). Patients with a SIS of 0, 1, 2, 3 or 4 had median OS of 10.5, 9.6, 5.2, 4.8 or 1.5 months respectively (p<0.001, log rank) (TIF 278 kb)
