# Supplementary material for: Combined systemic inflammation score (SIS) correlates with prognosis in patients with advanced pancreatic cancer receiving palliative chemotherapy
Source: J Cancer Res Clin Oncol. 2020 Aug 25;147(2):579–91. doi: 10.1007/s00432-020-03361-0 (PMC7817578; doi:10.1007/s00432-020-03361-0)
Supplement: Supplementary file 1 — Supplementary file1 (DOCX 12 kb) [file 432_2020_3361_MOESM1_ESM.docx]

| **Suppl. Table 1** Spearman-Rho correlation of SIR markers | | | | | | | | |
| --- | --- | --- | --- | --- | --- | --- | --- | --- |
| Marker | LMR | | NLR | | CRP | | mGPS | |
|  | R | p | R | p | R | p | R | p |
| grading | -0.121 | 0.211 | 0.08 | 0.413 | 0.211 | 0.021 | 0.217 | 0.017 |
| M1 | -0.225 | 0.019 | 0.283 | 0.003 | 0.394 | <0.001 | 0.328 | <0.001 |
| CA 19-9 | -0.208 | 0.049 | 0.386 | <0.001 | 0.171 | 0.086 | 0.195 | 0.050 |
| Abbreviations: LMR = lymphocyte-monocyte ratio; NLR = neutrophil-lymphocyte ratio; CRP = C-reactive protein; mGPS = modified Glasgow Prognostic Score; M1= metastatic disease | | | | | | | | |
