# Supplementary material for: Combined systemic inflammation score (SIS) correlates with prognosis in patients with advanced pancreatic cancer receiving palliative chemotherapy
Source: J Cancer Res Clin Oncol. 2020 Aug 25;147(2):579–91. doi: 10.1007/s00432-020-03361-0 (PMC7817578; doi:10.1007/s00432-020-03361-0)
Supplement: Supplementary file 2 — Supplementary file2 (DOCX 14 kb) [file 432_2020_3361_MOESM2_ESM.docx]

| **Suppl. Table 2** Impact of SIR markers in patients with mono- vs combination-therapy | | | | | | | | |
| --- | --- | --- | --- | --- | --- | --- | --- | --- |
|  | Monotherapy | | | | Combination-therapy | | | |
|  | months (95%CI) | p | HR (95%CI) | p | months (95%CI) | p | HR (95%CI) | p |
| all | 10.5 (8.2-12.9) | 0.669 |  |  | 10.8 (7.7-14.0) |  |  |  |
| LMR  >2.8  <2.8 | 9.8 (8.01-11.5)  7.9 (6.5-9.3) | 0.376 | 1.287 (0.735-2.255) | 0.378 | 12.8 (4.7-21.0) 6.1 4.0-8.2) | 0.089 | 2.067 (0.879-4.862) | 0.096 |
| NLR  <5  >5 | 9.6 (7.1-12.1)  5.1 (0.5-9.8) | 0.022 | 1.824 (1.080-3.080) | 0.025 | 10.8 (6.8-14.9)  4.8 (3.1-6.4) | 0.029 | 2.674 (1.068-6.699) | 0.036 |
| CRP  <5mg/dl  >5mg/dl | 11.2 (8.8-13.7)  4.7 (3.0-6.5) | 0.001 | 2.406 (1.422-4.073) | 0.001 | 11.3 (8.2-14.4)  1.1 (0.7-1.6) | <0.001 | 63.709 (7.294-556.459) | <0.001 |
| mGPS  0  >0 | 12.3 (8.8-15.9)  6.4 (3.3-9.6) | 0.002 | 2.120 (1.295-3.468) | 0.003 | 15.9 (14.3-17.5)  4.8 (3.7-5.8) | 0.018 | 2.579 (1.152-5.774) | 0.021 |
| Abbreviations: LMR = lymphocyte-monocyte ratio; NLR = neutrophil-lymphocyte ratio; CRP = C-reactive protein; mGPS = modified Glasgow Prognostic Score; M1= metastatic disease | | | | | | | | |
