# Supplementary material for: Combined systemic inflammation score (SIS) correlates with prognosis in patients with advanced pancreatic cancer receiving palliative chemotherapy
Source: J Cancer Res Clin Oncol. 2020 Aug 25;147(2):579–91. doi: 10.1007/s00432-020-03361-0 (PMC7817578; doi:10.1007/s00432-020-03361-0)
Supplement: Supplementary file 3 — Supplementary file3 (DOCX 14 kb) [file 432_2020_3361_MOESM3_ESM.docx]

| **Suppl. Table 3** Impact of SIR markers in patients with or without antibiotic therapy (N=148) | | | | | | | | |
| --- | --- | --- | --- | --- | --- | --- | --- | --- |
|  | with antibiotic therapy | | | | without antibiotic therapy | | | |
|  | months (95%CI) | p | HR (95%CI) | p | months (95%CI) | p | HR (95%CI) | p |
| all | 10.8 (7.4-14.2) | 0.415 | 1.158 (0.813-1.648) | 0.416 | 11.3 (9.4-13.3) |  |  |  |
| LMR  >2.8  <2.8 | N=33  10.8 (0.0-2.51)  9.5 (5.9-13.1) | 0.139 | 1.836 (0.812-4.150) | 0.145 | N=63  10.5 (6.0-15.1)  7.3 (5.2-9.4) | 0.030 | 1.891 (1.052-3.398) | 0.033 |
| NLR  <5  >5 | N=33  9.5 (3.0-16.0)  10.2 (2.7-17.7) | 0.582 | 1.229 (0.588-2.567) | 0.583 | N=63  10.5 (8.2-12.9)  4.0 (2.3-5.7) | <0.001 | 3.895 (2.110-7.192) | <0.001 |
| CRP  <5mg/dl  >5mg/dl | N=37  11.7 (9.1-14.3)  3.0 (0.4-5.6) | 0.031 | 2.235 (1.056-4.731) | 0.036 | N=66  10.5 (8.7-12.4)  4.0 (2.8-5.2) | <0.001 | 3.682 (1.985-6.830) | <0.001 |
| mGPS  0  >0 | N=37  10.8 (7.0-14.5)  7.9 (0.0-15.8) | 0.897 | 1.047 (0.518-2.117) | 0.897 | N=66  18.0 (8.3-27.7)  5.2 (3.7-6.8) | <0.001 | 3.111 (1.799-5.379) | <0.001 |
| Abbreviations: LMR = lymphocyte-monocyte ratio; NLR = neutrophil-lymphocyte ratio; CRP = C-reactive protein; mGPS = modified Glasgow Prognostic Score | | | | | | | | |
