# Supplementary figures and images for: Combined systemic inflammation score (SIS) correlates with prognosis in patients with advanced pancreatic cancer receiving palliative chemotherapy
Source: J Cancer Res Clin Oncol. 2020 Aug 25;147(2):579–91. doi: 10.1007/s00432-020-03361-0 (PMC7817578; doi:10.1007/s00432-020-03361-0)

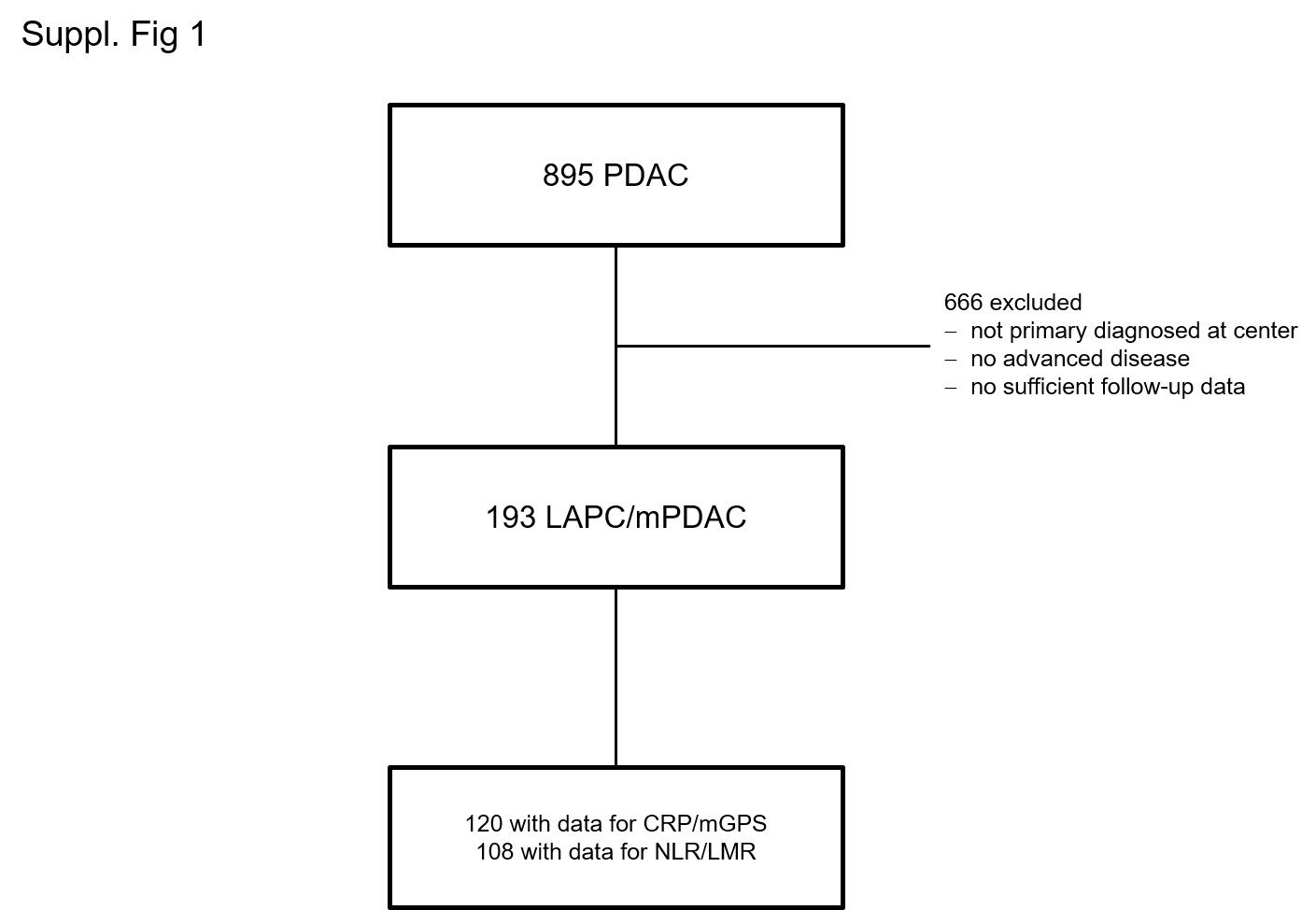

Supplement: Supplementary file 4 — Supplementary Figure 1 CONSORT Diagram. PDAC: pancreatic ductal adenocarcinoma; LAPC: locally advanced pancreatic cancer, mPDAC: metastatic pancreatic ductal adenocarcinoma; CRP: C-rective protein; mGPS: modified Glasgow Prognostic Score; NLR: neutrophil-lymphcyte ratio; LMR: lymphocyte-monocyte ratio (TIF 121 kb) [file 432_2020_3361_MOESM4_ESM.tif]

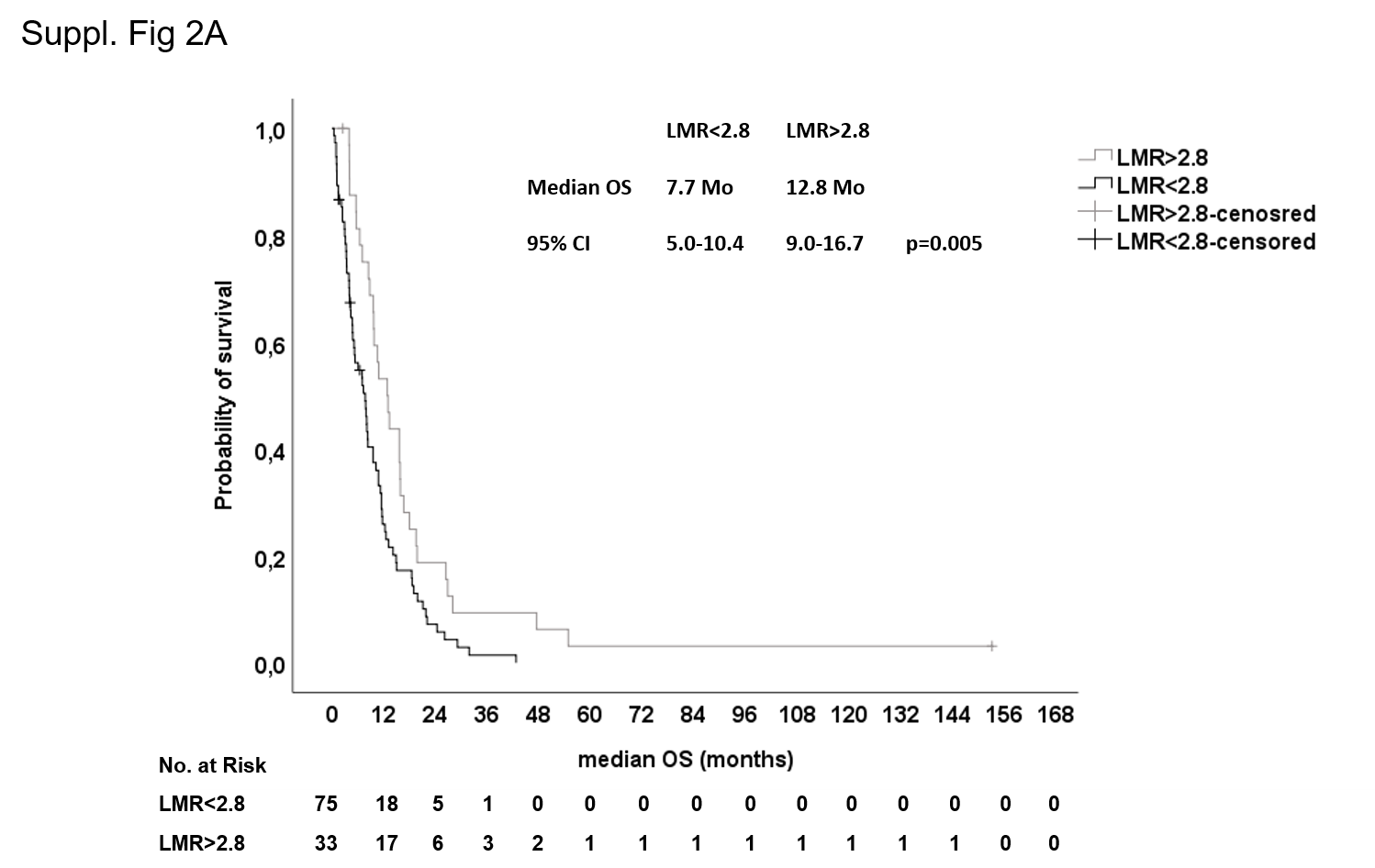

Supplement: Supplementary file 5 — Supplementary Figure 2 A, B Kaplan-Meier-plot for (A) overall survival (OS) and (B) Progression-free Survival (PFS) upon first-line palliative chemotherapy in relation to the lymphocyte-monocyte ratio (LMR). A) Patients with a LMR <2.8 had a median OS of 7.7 months and patients with a LMR>2.8 had a median OS of 12.8 months (p=0.005, log rank). B) Patients with a LMR <2.8 had a median PFS of 2.8 months and patients with a LMR>2.8 had a median PFS of 2.5 months (p=0.524, log rank) (TIF 216 kb) [file 432_2020_3361_MOESM5_ESM.tif]

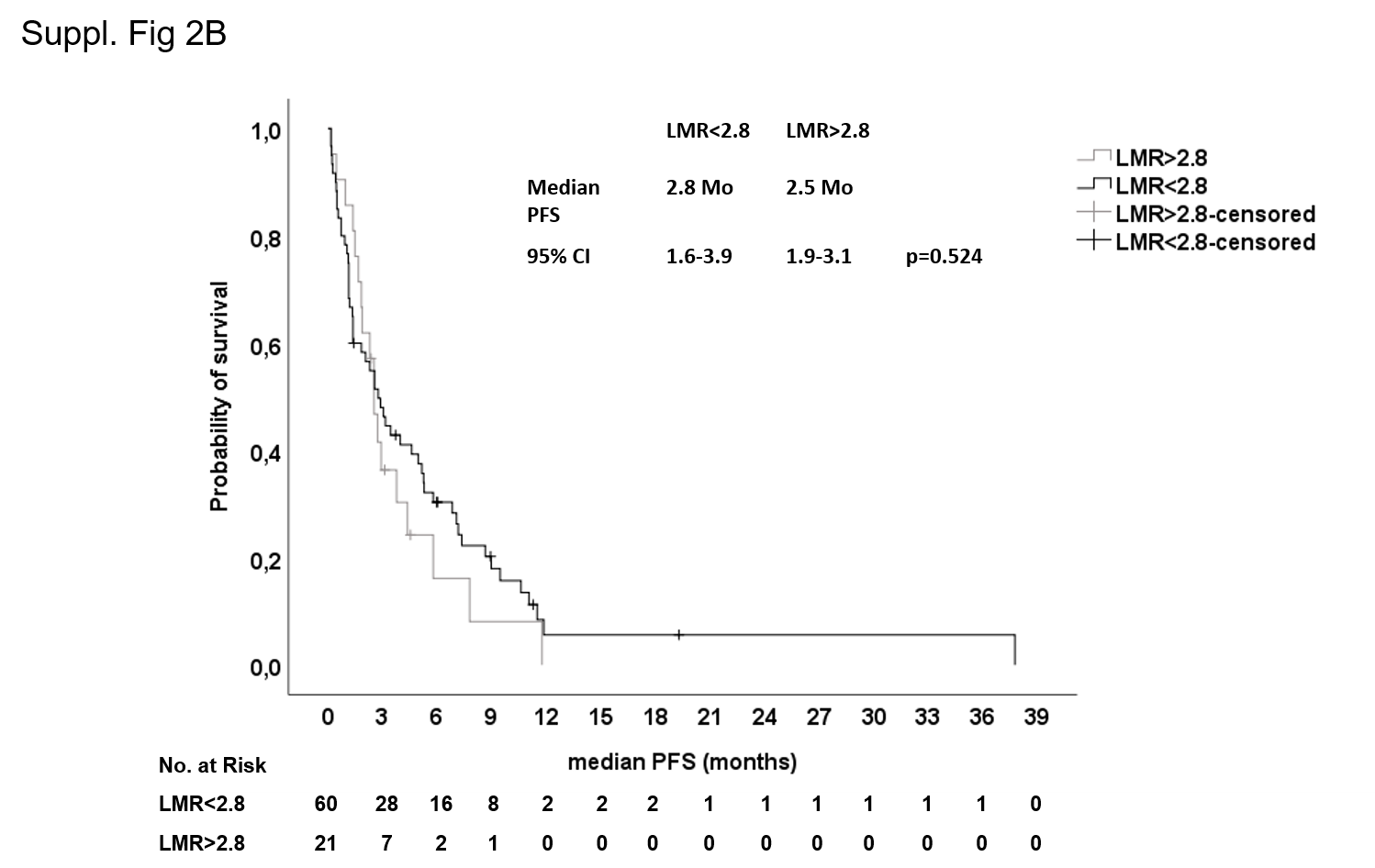

Supplement: Supplementary file 6 — Supplementary file6 (TIF 214 kb) [file 432_2020_3361_MOESM6_ESM.tif]

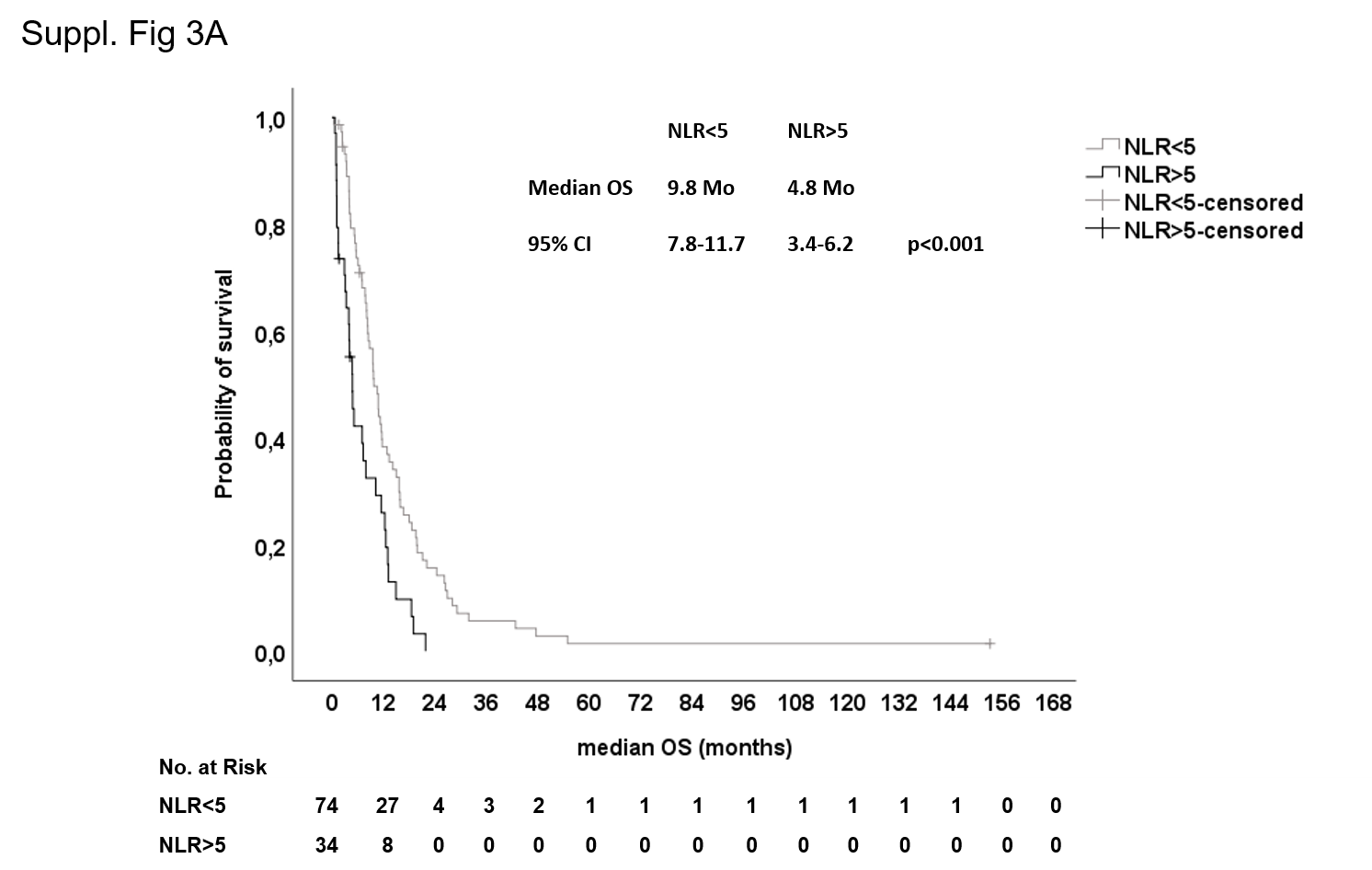

Supplement: Supplementary file 7 — Supplementary Figure 3 A, B Kaplan-Meier-plot for (A) overall survival (OS) and (B) Progression-free Survival (PFS) upon first-line palliative chemotherapy in relation to the neutrophil-lymphocyte ratio (NLR). A) Patients with a NLR>5 had a median OS of 4.8 months and patients with a NLR<5 had a median OS of 9.8 months (p<0.001, log rank). B) Patients with a NLR>5 had a median PFS of 2.6 months and patients with a NLR<5 had a median PFS of 2.9 months (p=0.044, log rank) (TIF 213 kb) [file 432_2020_3361_MOESM7_ESM.tif]

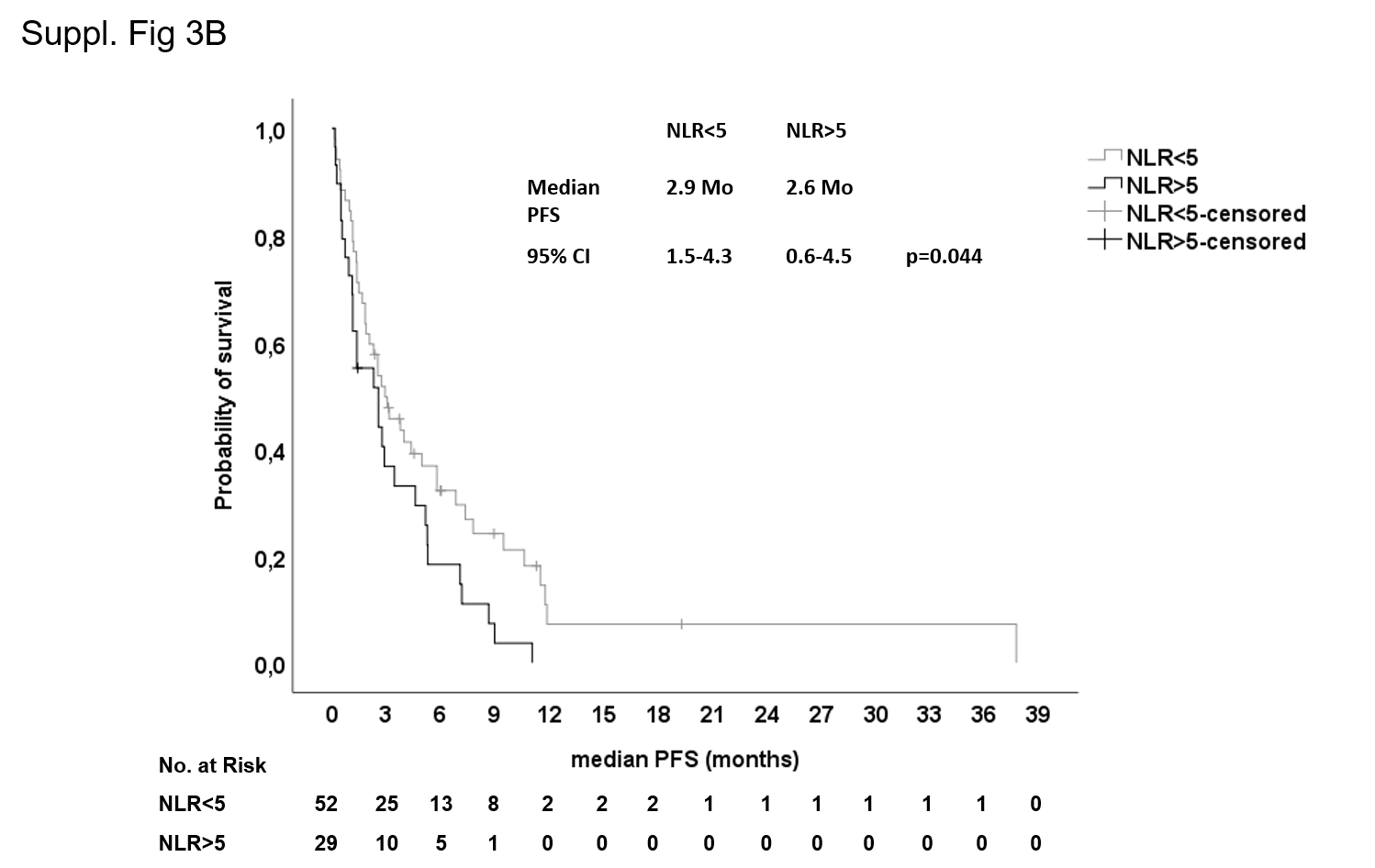

Supplement: Supplementary file 8 — Supplementary file8 (TIF 206 kb) [file 432_2020_3361_MOESM8_ESM.tif]

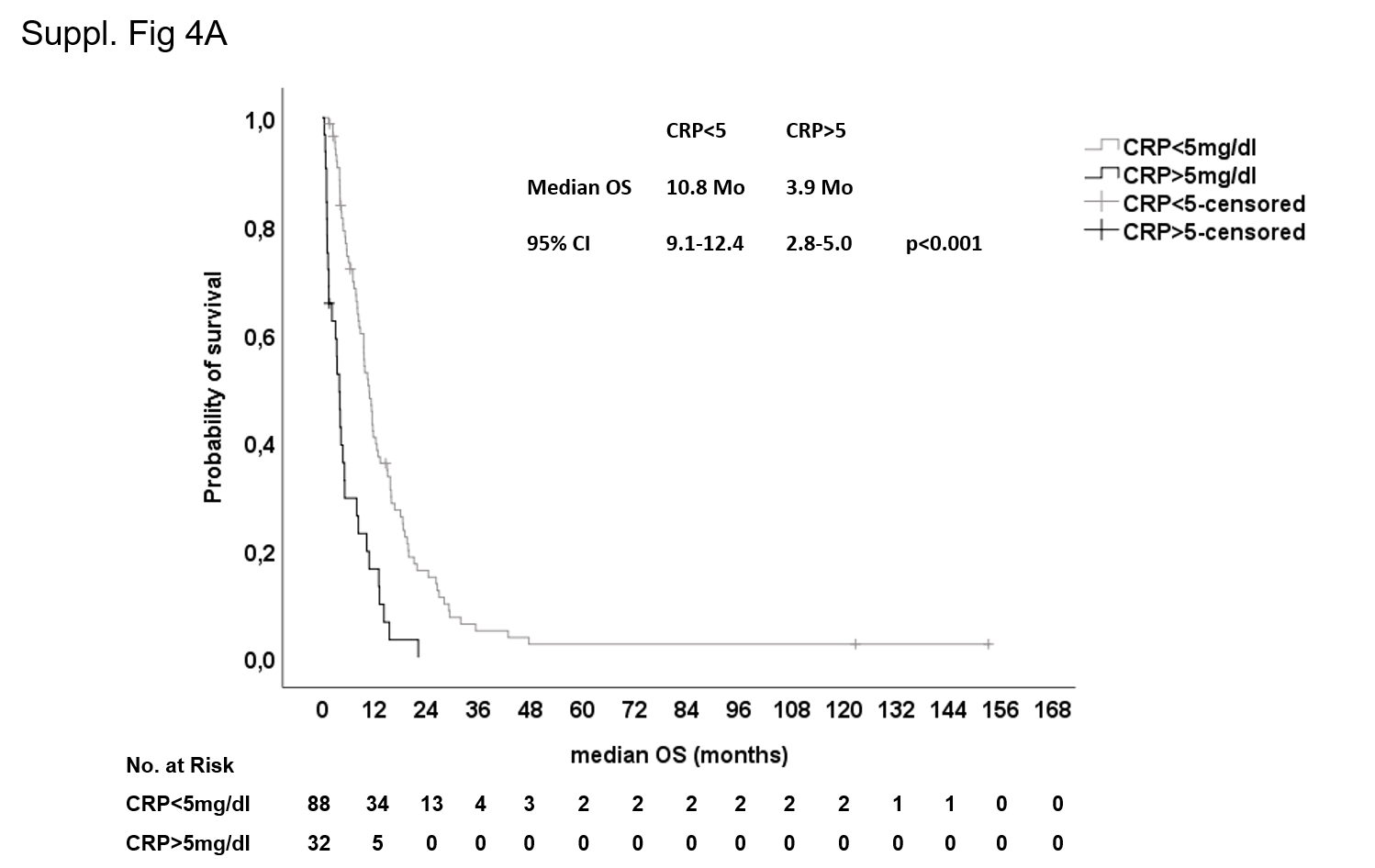

Supplement: Supplementary file 9 — Supplementary Figure 4 A, B Kaplan-Meier-plot for (A) overall survival (OS) and (B) Progression-free Survival (PFS) upon first-line palliative chemotherapy in relation to the C-reactive protein (CRP). A) Patients with a CRP>5 had a median OS of 3.9 months and patients with a CRP<5 had a median OS of 10.8 months (p<0.001, log rank). B) Patients with a CRP>5 had a median PFS of 1.2 months and patients with a CRP<5 had a median PFS of 3.2 months (p=0.036, log rank) (TIF 222 kb) [file 432_2020_3361_MOESM9_ESM.tif]

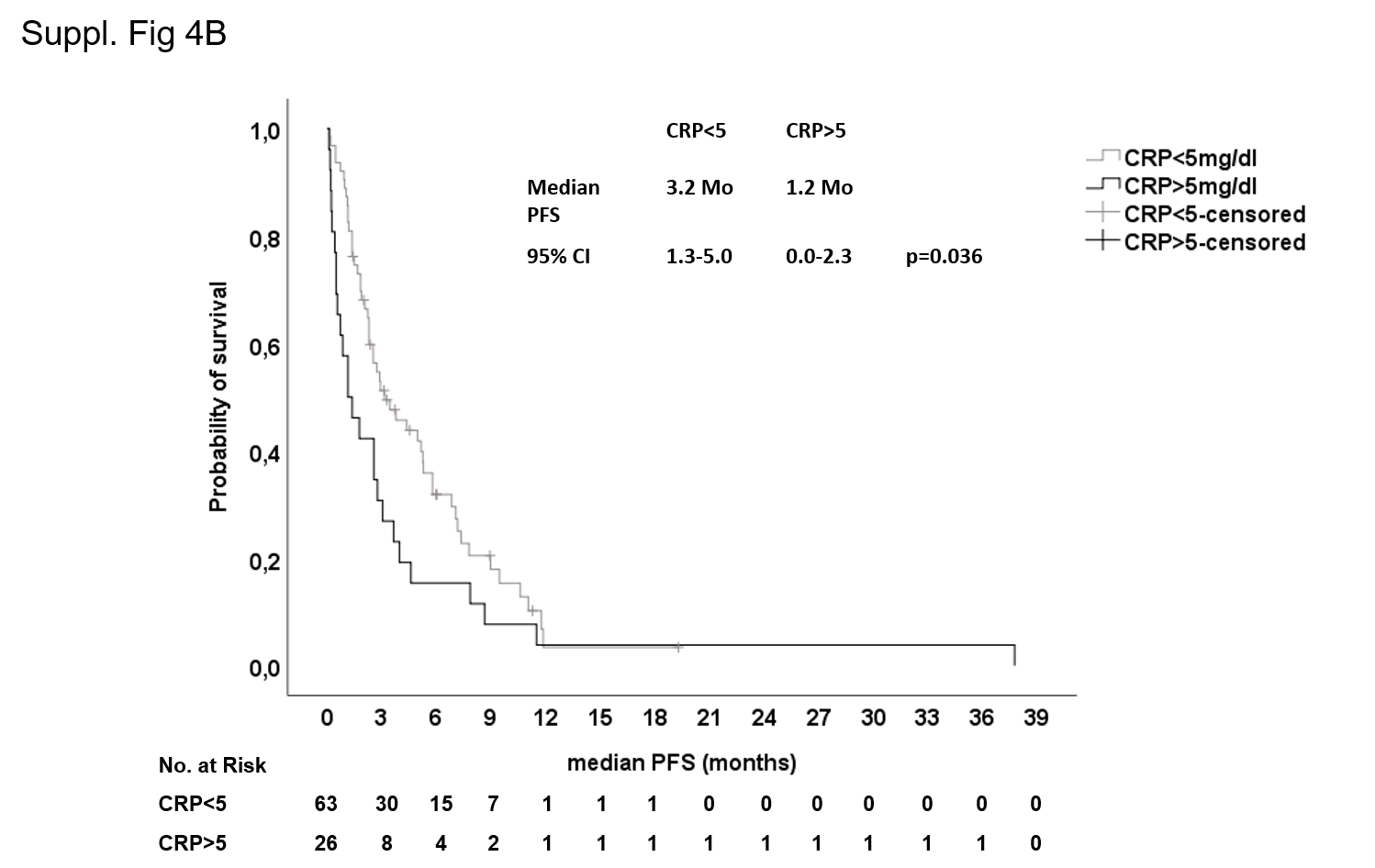

Supplement: Supplementary file 10 — Supplementary file10 (TIF 215 kb) [file 432_2020_3361_MOESM10_ESM.tif]

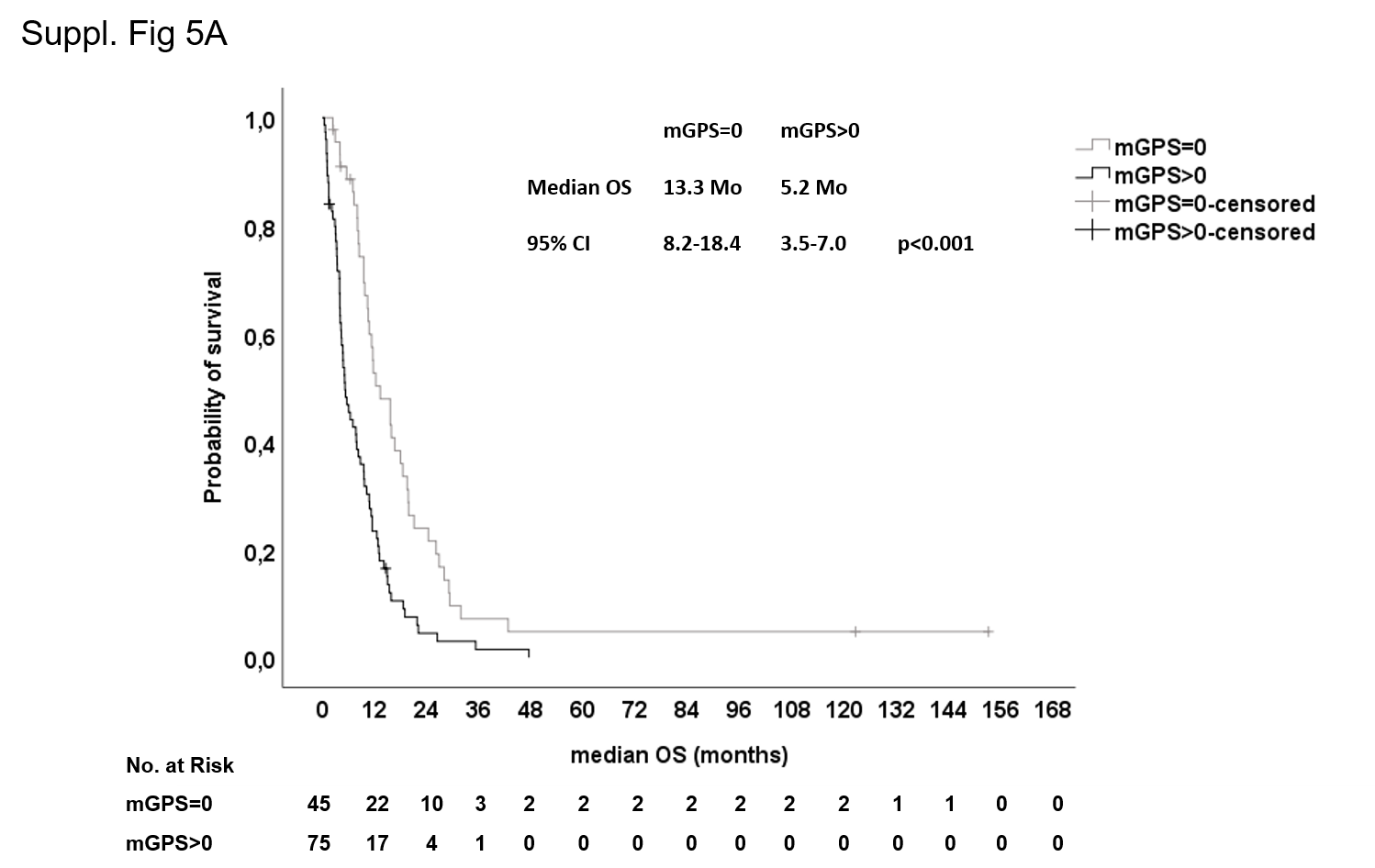

Supplement: Supplementary file 11 — Supplementary Figure 5 A, B Kaplan-Meier-plot for (A) overall survival (OS) and (B) Progression-free Survival (PFS) upon first-line palliative chemotherapy in relation to the modified Glasgow Prognostic Score (mGPS). A) Patients with a mGPS>0 had a median OS of 5.2 months and patients with a mGPS=0 had a median OS of 13.3 months (p<0.001, log rank). B) Patients with a mGPS>0 had a median PFS of 2.3 months and patients with a mGPS=0 had a median PFS of 5.8 months (p=0.030, log rank) (TIF 218 kb) [file 432_2020_3361_MOESM11_ESM.tif]

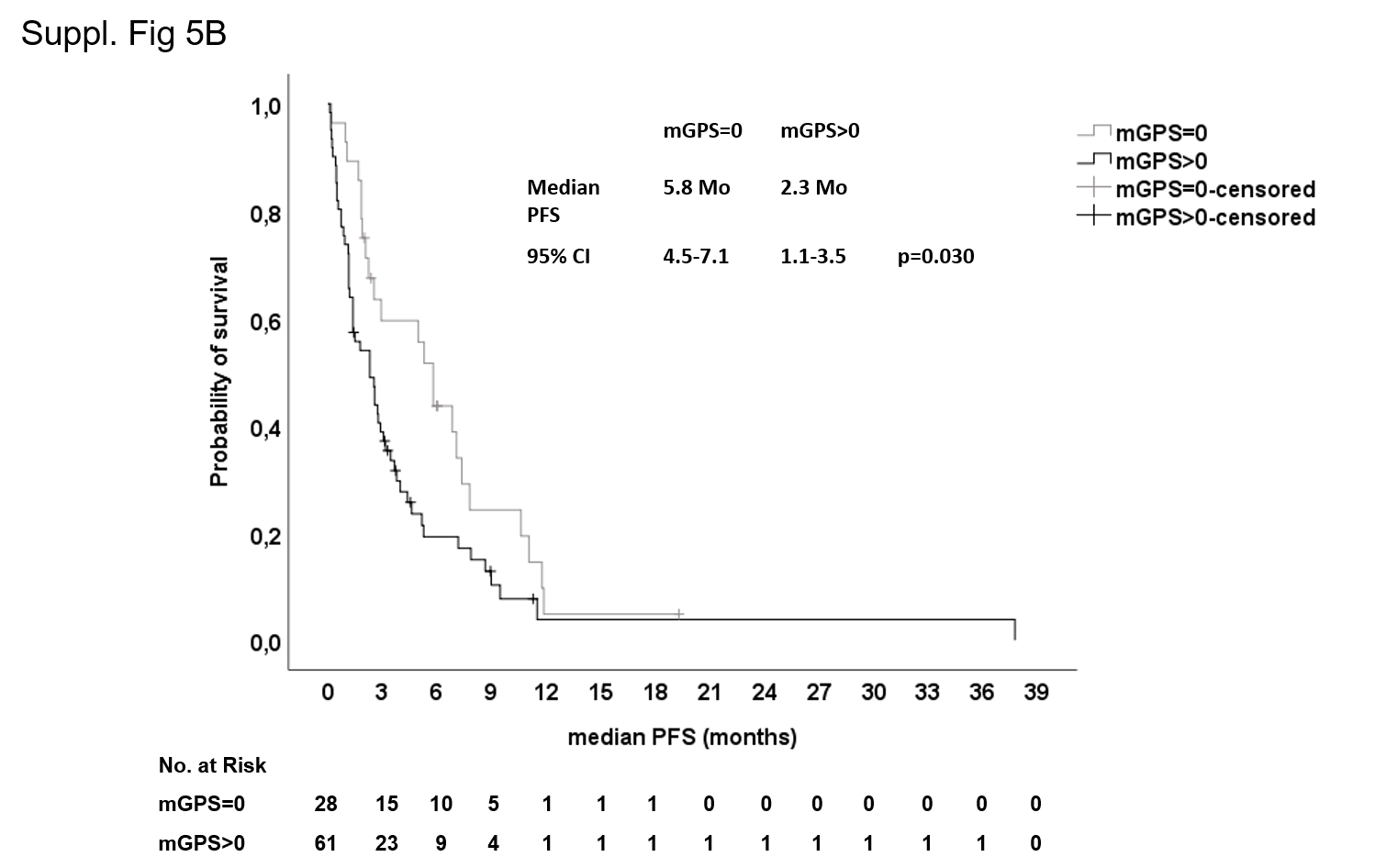

Supplement: Supplementary file 12 — Supplementary file12 (TIF 212 kb) [file 432_2020_3361_MOESM12_ESM.tif]

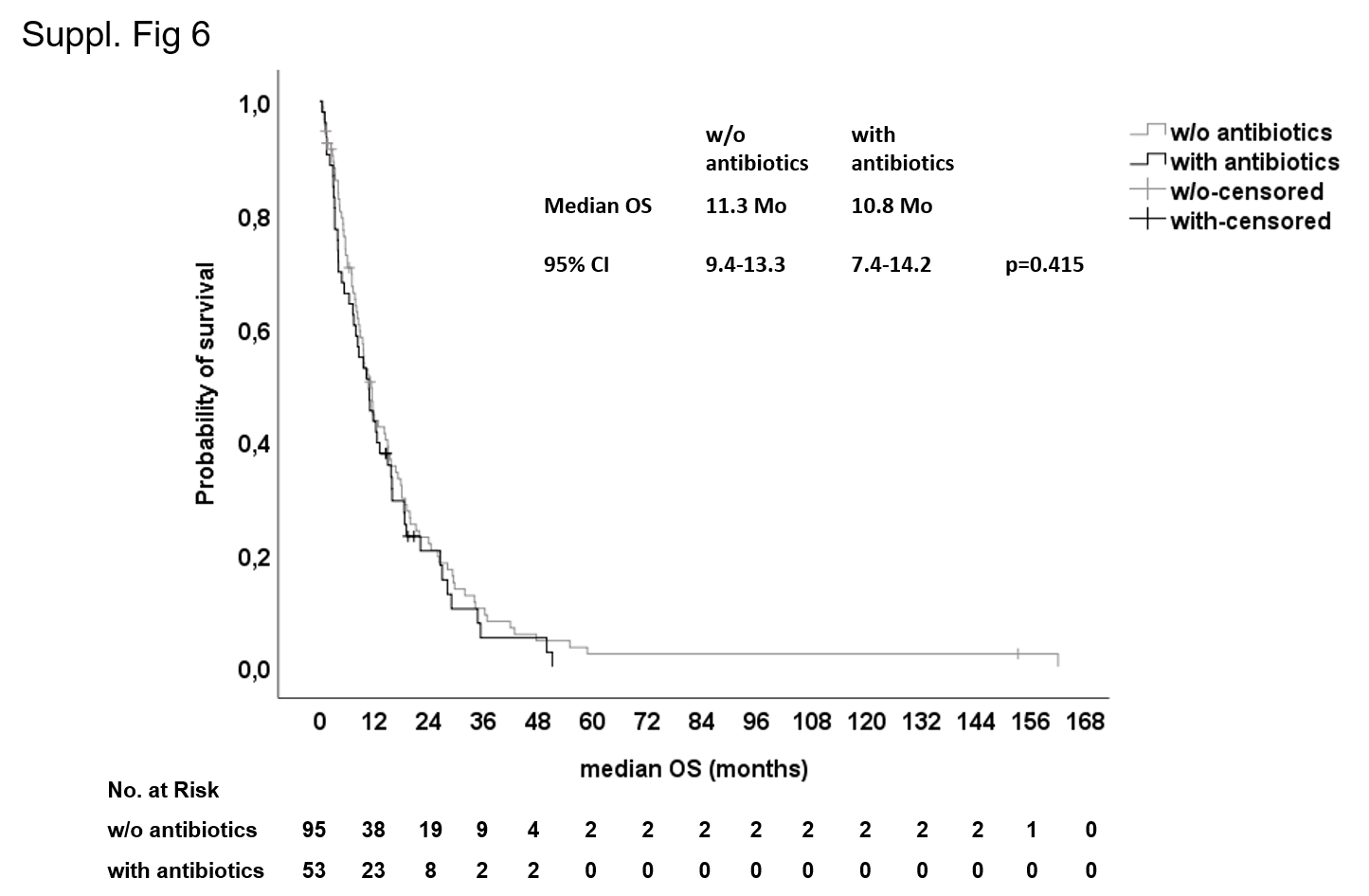

Supplement: Supplementary file 13 — Supplementary Figure 6 Kaplan-Meier-plot for overall survival (OS) upon first-line palliative chemotherapy in relation to the use of antibiotics. Patients, which received antibiotics had a median OS of 10.8 months and patients without antibiotics (w/o) had a median OS of 11.3 months (p=0.415, log rank) (TIF 224 kb) [file 432_2020_3361_MOESM13_ESM.tif]

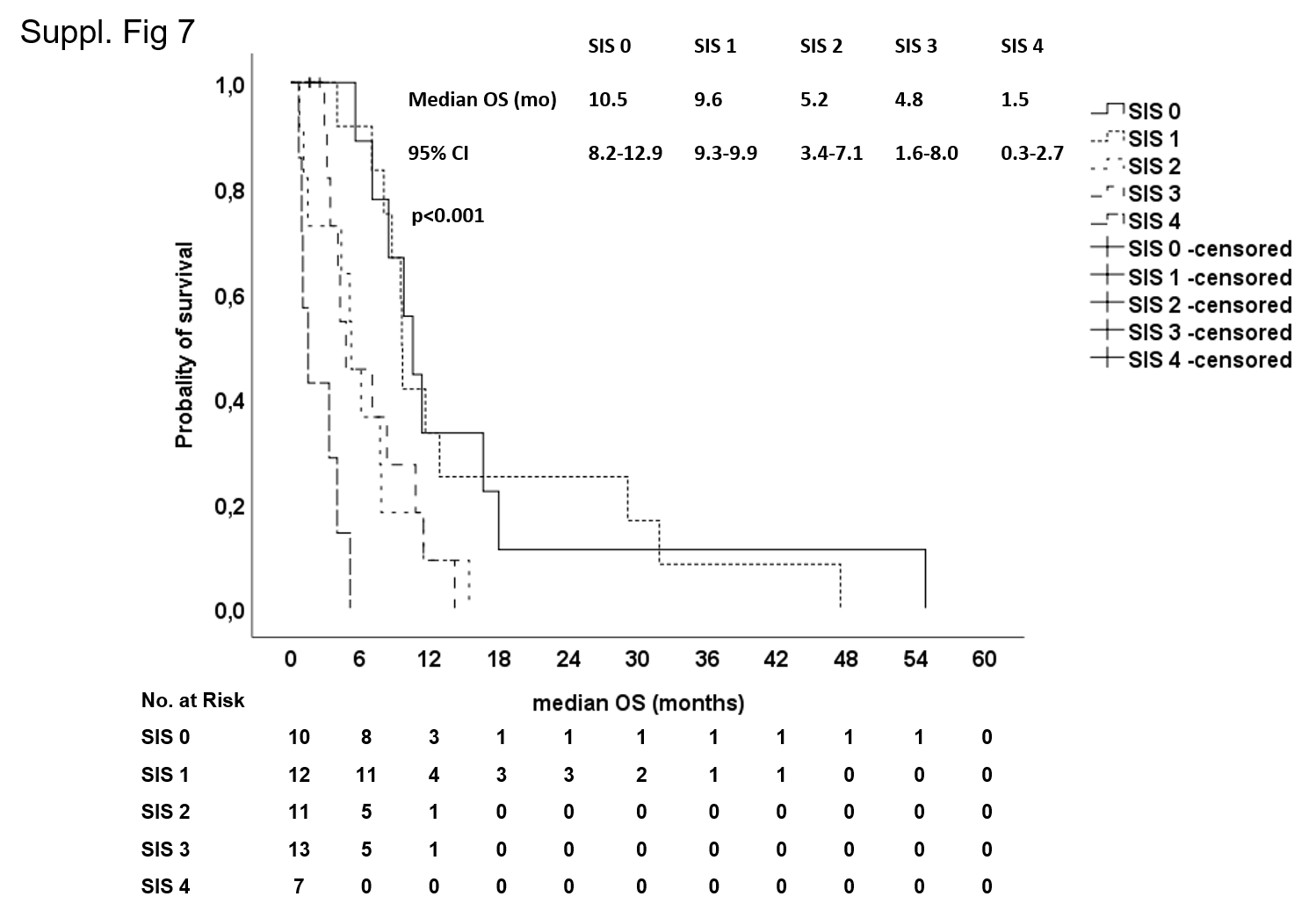

Supplement: Supplementary file 14 — Supplementary Figure 7 Kaplan-Meier-plot for overall survival (OS) in patients with metastatic disease, which were not treated with antibiotics upon first-line palliative chemotherapy stratified for the Systemic Inflammation Score (SIS). Patients with a SIS of 0, 1, 2, 3 or 4 had median OS of 10.5, 9.6, 5.2, 4.8 or 1.5 months respectively (p<0.001, log rank) (TIF 278 kb) [file 432_2020_3361_MOESM14_ESM.tif]
